# Community socioeconomic deprivation and SARS-CoV-2 infection risk: findings from Portugal

**DOI:** 10.1093/eurpub/ckab192

**Published:** 2021-11-11

**Authors:** João Paulo M Magalhães, Ana Isabel Ribeiro, Constantino P Caetano, Rita Sá Machado

**Affiliations:** 1 Unidade de Saúde Pública, ACES Porto Oriental, Administração Regional de Saúde do Norte, Porto, Portugal; 2 Divisão de Epidemiologia e Estatística, Direção-Geral da Saúde, Lisboa, Portugal; 3 EPIUnit—Instituto de Saúde Pública da Universidade do Porto, Porto, Portugal; 4 Departamento Ciências da Saúde Pública e Forenses, e Educação Médica, Faculdade de Medicina da Universidade do Porto, Porto, Portugal; 5 Laboratório para a Investigação Integrativa e Translacional em Saúde Populacional (ITR), Porto, Portugal; 6 Departamento de Epidemiologia, Instituto Nacional de Saúde Doutor Ricardo Jorge, Lisboa, Portugal; 7 Unidade de Saúde Pública, ACES Almada/Seixal, Administração Regional de Saúde Lisboa e Vale do Tejo, Lisboa, Portugal

## Abstract

**Background:**

Socioeconomic differences have been observed in the risk of acquiring infectious diseases, but evidence regarding SARS-CoV-2 remains sparse. Hence, this study aimed to investigate the association between SARS-CoV-2 infection risk and socioeconomic deprivation, exploring whether this association varied according to different phases of the national pandemic response.

**Methods:**

A cross-sectional study was conducted. Data routinely collected for patients with a laboratorial result recorded in SINAVE^®^, between 2 March and 14 June 2020, were analysed. Socioeconomic deprivation was assessed using quintiles of the European Deprivation Index (Q1-least deprived to Q5-most deprived). Response phases were defined as before, during and after the national State of Emergency. Associations were estimated using multilevel analyses.

**Results:**

The study included 223 333 individuals (14.7% were SARS-CoV-2 positive cases). SARS-CoV-2 infection prevalence ratio increased with deprivation [PR(Q1)=Ref; PR(Q2)=1.37 (95% CI 1.19–1.58), PR(Q3)=1.48 (95% CI 1.26–1.73), PR(Q4)=1.73 (95% CI 1.47–2.04), PR(Q5)=2.24 (95% CI 1.83–2.75)]. This was observed during the State of Emergency [PR(Q5)=2.09 (95% CI 1.67–2.62)] and more pronounced after the State of Emergency [PR(Q5)= 3.43 (95% CI 2.66–4.44)].

**Conclusion:**

The effect of socioeconomic deprivation in the SARS-CoV-2 infection risk emerged after the implementation of the first State of Emergency in Portugal, and became more pronounced as social distancing policies eased. Decision-makers should consider these results when deliberating future mitigation measures.

## Introduction

The World Health Organization (WHO) stated that emerging diseases are one of the challenges for the next decade and century.^1^ Demographic, epidemiologic and technological transitions, framed within the global economic context, create the conditions to transform a localized outbreak into a pandemic.^2^^,^^3^ The infection caused by the severe acute respiratory syndrome coronavirus 2 (SARS-CoV-2) was first recognized on 31 December 2019 by the WHO as a cluster of unknown origin in Wuhan, China.^4^^,^^5^ The distribution of health outcomes from coronavirus disease 2019 (COVID-19) in the population is not yet fully known, but there have been differences observed by socioeconomic groups, which can compromise the responsiveness of the health system in each country.^4^

When facing an emerging disease, understanding transmission patterns of the agent and their enablers, such as contemporary global mobility, is essential.^2^^,^^6^ In a person-to-person respiratory disease, active community transmission and the risk of infection are determined, among other factors, by population density, workplace, location of entry points, public transportation, and social and economic determinants, that influence individual behaviour and environmental exposures.^4^^,^^7^ Social determinants influence the likelihood of illness the most and are characterized by the political, economic and societal context and, consequently, the individual position held by each.^7–9^ These processes dictate the capital and social cohesion of a community and society, and determine the adoption of salutogenic individual behaviours, as well as the environment in which the groups are inserted.^10^^,^^11^ Additionally, the degree to which social factors determine the spread of and infection with an emerging disease might be influenced by the stringency of the public health response. On the one hand, in the beginning of the epidemic in Portugal, most reported cases were imported and associated with national individuals, mainly less deprived populations returning from international events (e.g. Milan fashion fairs), Carnival and snow resort holidays, many of them located in Northern Italy. On the other hand, after community transmission is established, a communicable disease might disproportionally affect the most deprived populations due to their greater exposure to the infectious agent.

In Portugal, the first SARS-CoV-2 confirmed case was identified on 2 March 2020 and at an early stage, the potential for transmission remained high, with an R(*t*) = 2.07 until 3 March 2020.^^12^,^13^^ The first national State of Emergency was declared on 19 March 2020 and was renewed biweekly until 2 May 2020.^13^ The State of Emergency legislative measures enforced the closure of international borders, and the suspension of non-essential services and events. Residents could only leave their homes to shop for basic goods, to take care of vulnerable people, to walk their dogs or dispose of daily residuals, and to go to work (limited to those with essential jobs). These measures, complemented by epidemiological surveillance, early case detection and contact tracing, reduced the R(*t*) to below or close to 1 from 2 April 2020 until mid-June.^12^^,^^14^

Although the presence of socioeconomic differences in the risk of SARS-COV-2 infection has been explored in a few recent studies.^4^^,^^15^^,^^16^ to the best of our knowledge, no studies have investigated the modification effect of the lockdown and mitigation measures in the association between socioeconomic deprivation and SARS-CoV-2 infection. Many disadvantaged individuals tend to be employed in essential sectors, not allowing for working from home modalities during lockdown.^17^ Thus, we hypothesize that socioeconomic differences in the risk of SARS-CoV-2 infection may have increased over time, particularly during the State of Emergency.

This study aimed to investigate the association between the risk of SARS-CoV-2 infection and socioeconomic deprivation in Portugal, and to explore whether this association varied according to the different phases of national response to the pandemic.

## Methods

A cross-sectional study was carried out with individuals notified as suspected cases of SARS-CoV-2 infection in Portugal, between 14 February and 14 June 2020.

Suspected cases of SARS-CoV-2 infection registered in the clinical notification in the National Epidemiological Surveillance System (SINAVE^®^) were included in the current study. Cases were excluded if their clinical notification of SARS-CoV-2 infection did not have information regarding the parish of occurrence.

In Portugal, SINAVE^®^ is governed by Decree Law no. 81/2009, 21 August 2009, which states (paragraph 4, article 20) that the processing of personal data when essential for the purposes of surveillance, risk assessment and management of Public Health, must be carried out by qualified health professionals, led by health authorities. Thus, no additional ethical approval was necessary for this study since secondary data was used.

The exposure variable was socioeconomic deprivation, measured using the European Deprivation Index (EDI), which was previously validated for the Portuguese territory.^18^ The EDI was created at the parish level (*n* = 3091), which has a population ranging between 31 and 66 250 and is the smallest geographical units of health data dissemination in Portugal, reducing ecological bias. The EDI was grouped into quintiles with a balanced population distribution, where the first quintile (least deprived) included 2 185 289 inhabitants (20.7% of the national population); the second, 2 199 410 (20.8%); the third, 2 189 526 (20.7%); the fourth, 2 097 658 (19.9%); and the fifth (most deprived), 1890.244 (17%). The EDI level was assigned to each individual according to the parish of occurrence, defined on SINAVE as ‘place of infection’.^19^ The outcome variable was the laboratory result for SARS-CoV-2 infection, obtained through clinical notification, which was used as a binary variable (positive and negative).

The remaining variables were selected and categorized considering the geodemographic, clinical and epidemiological factors that could influence the causal path between exposure and outcome, and scientific evidence. The following effect-modifying variables were considered: time (days) between the onset of symptoms and the diagnosis (hereafter named delay), national pandemic response phase when the case was registered [pre-State of Emergency (14 February to 22 March 2020), State of Emergency (23 March to 2 May 2020) and post-State of Emergency (3 May to 14 June 2020)], and an identifiable epidemiological link (yes/no). The following confounding variables were also considered: age, sex, comorbidities, health region, typology of urban area and population density. The response phases were defined considering the measures applied to the entire Portuguese population, excluding, for example, local lockdowns.

A multilevel analysis was performed in three steps (model assumptions are provided in Supplementary figures S1 and S2, and table S1), considering two clustering levels (tiers 1 and 2), to calculate prevalence ratios (PRs) and 95% confidence intervals (CI). The models were carried out using a simple association between tier 2 SES and tier 1 laboratory result (Model 0), and then were successively adjusted for tiers 1 sex and age (Model 1), tiers 2 typology of urban area and population density (Model 2) and, finally, tier 2 health region (Model 3). A Model 3 multilevel analysis was also conducted considering response phase, and both the North and Lisbon and Tagus Valley (LTV) health regions (model assumptions are provided in Supplementary tables S2 and S3).

Due to missing data in clinical factors, and potential inconsistency between the place of occurrence and residence parish, a sensitivity analysis was carried out. A multilevel analysis including tier 1 comorbidities and a concordance analysis for parishes was performed. The statistical software *OpenEpi* was used for power calculation and R version 4.0.3 was used for multilevel analysis (packages ‘stats’ and ‘lme4’).^20–22^

## Results

A total of 223 333 suspected cases were included in the study [after excluding 26 cases (0.01%) due to lack of parish of occurrence], from which 32 784 tested positive for SARS-CoV-2 infection.

The mean age of all cases included, regardless of the SARS-CoV-2 laboratory result, decreased from least deprived (Q1) to most deprived (Q5) (51–43 years; *P* < 0.05). The proportion of all cases increased from Q1 to Q5 from 0–19 years of age until 40–49 years and decreased in the remaining age groups. Females had the highest number of total cases in all quintiles (*P* < 0.05) (table 1). The distribution of all cases differed according to the health region (*P* < 0.05), with a greater proportion in Q1, Q2 and Q3 in the North, Q1 in the Centre, Q4 and Q5 in the LTV region, and Q3 in *Alentejo*. Regarding the typologies of urban areas and population density, Q1 had the most uniform distribution, while the remaining quintiles had a higher proportion of total cases in predominantly urban areas (especially in Q5), and the median population density increased from Q1 to Q5 (*P* < 0.05). Comorbidities were more frequent among all cases from Q5 (28.5%), and differences were observed between quintiles in all diseases (*P* < 0.05) (table 2). Total cases in Q1 had a shorter delay in accessing healthcare compared to Q5 (4.4 days vs. 5.0 days, respectively), and the proportion of total cases with an unknown epidemiological link was higher in Q5 (21.2%). Finally, the distribution by pandemic response phases was not uniform (*P* < 0,05), as the State of Emergency (54.2%) and post-State of Emergency (35.6%) had most of the total cases. In the pre-State of Emergency, the total cases occurred mostly in Q2 and Q3; in the State of Emergency mainly in Q1, Q2 and Q3; and in the post-State of Emergency, cases were concentrated in Q5.

**Table 1:** Absolute and relative frequencies of SARS-CoV-2 cases, by geodemographic factors, according to five quintiles of socioeconomic deprivation

Socioeconomic deprivation (quintiles)	Q1 [<deprived]	Q2	Q3	Q4	Q5 [>deprived]	Total
	(*n* = 44 901)	(*n* = 44 432)	(*n* = 44 763)	(*n* = 44 619)	(*n* = 44 618)	(*n* = 223 333)
Geodemographic factors						
Age (years)						
Mean (IQR)	51 (33–72)	48 (31.0–67.0)	46 (29–64)	44 (28–62)	43 (28–61)	46 (29–65)
Age groups [*n* (%)]						
0–19	4712 (10.5)	5546 (12.4)	6321 (14.0)	6442 (14.6)	6138 (13.8)	29 159 (13.1)
20–29	4478 (10.0)	4939 (11.0)	5226 (11.6)	5651 (12.8)	6461 (14.5)	26 755 (12.0)
30–39	6062 (13.6)	6454 (14.4)	6554 (14.5)	6734 (15.3)	7247 (16.2)	33 051 (14.8)
40–49	6411 (14.4)	6699 (14.9)	7144 (15.8)	6754 (15.3)	6891 (15.4)	33 899 (15.2)
50–59	6117 (13.7)	6274 (14.0)	6392 (14.1)	6019 (13.7)	5813 (13.0)	30 615 (13.7)
60–69	4833 (10.8)	4756 (10.6)	4762 (10.5)	4395 (10.0)	4242 (9.5)	22 988 (10.3)
70–79	4429 (9.9)	4055 (9.0)	3814 (8.4)	3508 (8.0)	3528 (7.9)	19 334 (8.7)
80+	7631 (17.1)	6116 (13.6)	4975 (11.0)	4528 (10.3)	4282 (9.6)	27 532 (12.3)
Sex [*n* (%)]						
Female	26 175 (58.6)	26 025 (58.0)	26 295 (58.2)	25 155 (57.1)	24 924 (55.9)	12 8574 (57.6)
Male	18 498 (41.4)	18 814 (42.0)	18 893 (41.8)	18 876 (42.9)	19 678 (44.1)	94 759 (42.4)
Health region [*n* (%)]						
North	21 876 (49.0)	21 380 (47.7)	23 073 (51.1)	18 271 (41.5)	4793 (10.7)	89 393 (40.0)
Centre	15 642 (35.0)	8769 (19.6)	4821 (10.7)	787 (1.8)	291 (0.7)	30 310 (13.6)
LTV	6228 (13.9)	11 823 (26.4)	13 052 (28.9)	19 840 (45.1)	29 168 (65.4)	80 111 (35.9)
*Alentejo*	536 (1.2)	2368 (5.3)	3940 (8.7)	2699 (6.1)	1365 (3.1)	10 908 (4.9)
*Algarve*	24 (0.1)	191 (0.4)	40 (0.1)	1275 (2.9)	8775 (19.7)	10 305 (4.6)
*Açores*	337 (0.8)	166 (0.4)	154 (0.3)	58 (0.1)	30 (0.1)	745 (0.3)
*Madeira*	30 (0.1)	142 (0.3)	108 (0.2)	1101 (2.5)	180 (0.4)	1561 (0.7)
Urban areas [*n* (%)]						
PUA	21 119 (47.3)	29 606 (66.0)	38 469 (85.1)	36 665 (83.3)	39 118 (87.7)	164 977 (73.9)
MUA	12 417 (27.8)	8340 (18.6)	3247 (7.2)	3456 (7.8)	1471 (3.3)	28 931 (13.0)
PRA	11 137 (24.9)	6893 (15.4)	3472 (8.9)	3910 (8.9)	4013 (9.0)	29 425 (13.2)
Population density (habitants/km^2^)	213.6	361.9	815.6	1512.4	3020.1	690.6
Median (IQR)	(76.4–616.9)	(141.8–1182.1)	(391.5–4069.6)	(415.1–3372.8)	(596.4–6690.5)	(184.0–2878.6)

PUA, predominantly urban areas; MUA, mainly urban areas; PRA, predominantly rural areas.

**Table 2:** Absolute and relative frequencies of SARS-CoV-2 cases, by clinical and epidemiological factors, according to five quintiles of socioeconomic deprivation

Socioeconomic deprivation (quintiles)	Q1 [<deprived]	Q2	Q3	Q4	Q5 [>deprived]	Total
	(*n* = 44 901)	(*n* = 44 432)	(*n* = 44 763)	(*n* = 44 619)	(*n* = 44 618)	(*n* = 223 333)
Clinical factors						
Comorbidities [*n* (%)]						
No	13 709 (30.7)	15 169 (33.8)	16 520 (36.6)	16 406 (37.3),	17 468 (39.2),	79 272 (35.5)
Yes	10 419 (23.3)	11 534 (25.7)	11 553 (25.6)	11 052 (25.1)	12 725 (28.5)	57 283 (25.6)
Missing	20 545 (46.0)	18 136 (40.4)	17 115 (37.9)	16 573 (37.6)	14 409 (32.3)	86 778 (38.9)
Epidemiological factors						
Delay (days)						
Median (IQR)	4.4 (0.0–5.0)	5.0 (1.0–6.0)	5.0 (1.0–6.0)	5.1 (1.0–6.0)	5.0 (1.0–6.0)	4.9 (1.0–6.0)
Epidemiology link [*n* (%)]No	17 960 (40.2)	18 353 (40.9)	19 000 (42.0)	17 972 (40.8)	19 477 (43.7)	92 762 (41.5)
Yes	6756 (15.1)	8225 (18.3)	9035 (20.0)	9245 (21.0)	9463 (21.2)	42 724 (19.1)
Missing	19 957 (44.7)	18 261 (40.7)	17 153 (38.0)	16 814 (38.2)	15 662 (35.1)	87 847 (39.3)
Response phase [*n* (%)]						
Pre-State of Emergency	3836 (8.6)	5223 (11.6)	5286 (11.7)	4552 (10.3)	3993 (9.0)	22 890 (10.2)
State of Emergency	25 125 (56.2)	25 041 (55.8)	25 245 (55.9)	24 103 (54.7)	21 531 (48.3)	121 045 (54.2)
Post-State of Emergency	15 712 (35.2)	14 575 (32.5)	14 657 (32.4)	15 376 (34.9)	19 078 (42.8)	79 398 (35.6)

To identify and compare the factors for SARS-CoV-2 infection, before desegregating by EDI quintiles, the total number of negative and positive SARS-CoV-2 infection cases (Supplementary table S4) were analysed. The positive SARS-CoV-2 infection cases were older [48 years (interquartile range, IQR 32–64) vs. 46 (IQR 29–65)] and more likely to be male [PR = 1.07 (95% CI 1.02–1.12)]. Positive cases occurred more frequently in predominantly urban areas [PR = 0.90 (95% CI 0.83–0.97)] and in higher population density areas [1802.6 (IQR 448.6–4820.7) vs. 616.9 (IQR 150.8–2734.6)]. Regarding clinical factors, there were no differences in the proportion of comorbidities reported [PR = 1.01 (95% CI 0.98–1.04)] and there were no statistically differences in delay to access healthcare services [PR = 1.00 (95% CI 0.99–1.01)].

**Figure 1:**
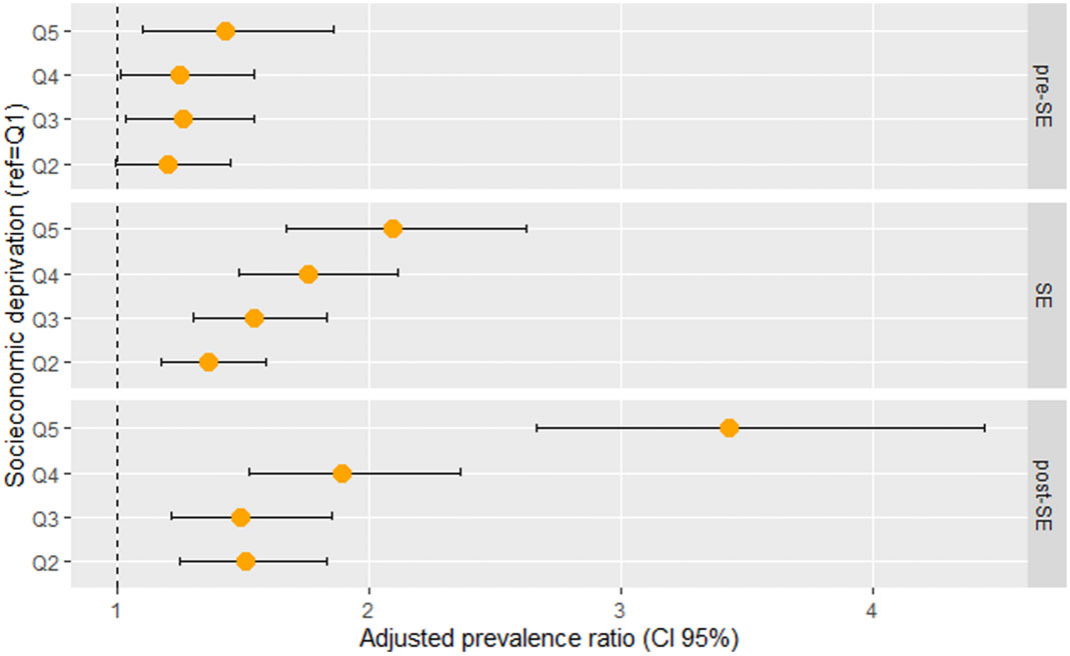
Adjusted PRs between socioeconomic deprivation, in quintiles, and SARS-CoV-2 infection, by response phase Model adjusted for age, sex, typology of urban areas, population density and health region SE, State of Emergency

Considering all response phases, the multilevel analysis (Supplementary table S5) showed a statistically significant increasing gradient of SARS-CoV-2 infection risk from least deprived to most deprived. With the exception of Model 2, Q5 cases were at higher risk of infection and, in the fully adjusted model, PRs of 1.37 (95% CI 1.19–1.58), 1.48 (95% CI 1.26–1.73), 1.73 (95% CI 1.47–2.04) and 2.24 (95% CI 1.83–2.75) were observed for each EDI (Q2, Q3, Q4, Q5, respectively), when compared to Q1. Analyses by response phase to the pandemic (figure 1 and Supplementary table S6) showed an increasing gradient with socioeconomic deprivation, from Q1 to Q5, except in the pre-State of Emergency [State of Emergency, PR(Q5) = 2.09 (95% CI 1.67–2.62) and post-State of Emergency, PR(Q5) = 3.43 (95% CI 2.66–4.44)].

**Figure 2:**
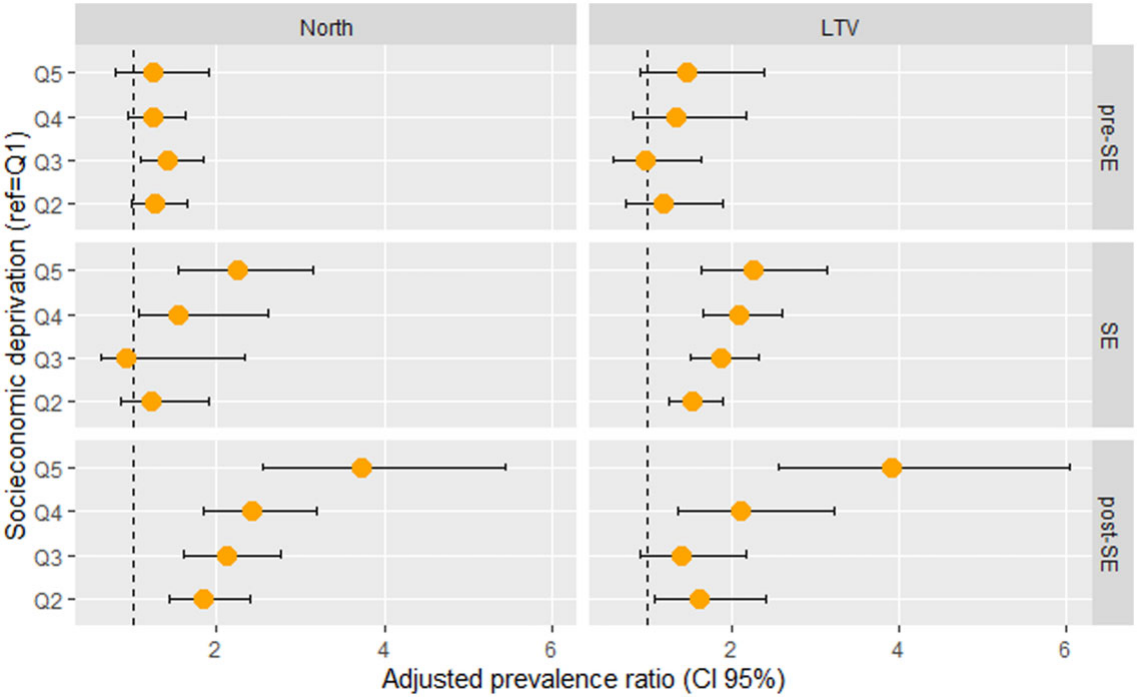
Adjusted PRs between socioeconomic deprivation, in quintiles, and SARS-CoV-2 infection, by response phase Model adjusted for age, sex, typology of urban areas and population density SE, State of Emergency

Due to the higher number of cases reported in the North and LTV regions, the multilevel analysis was conducted in these regions by pandemic response phases (figure 2 and Supplementary table S6). During the pre-State of Emergency, no statistically significant differences were observed between quintiles, while a statistically increasing gradient was observed in both areas during the State of Emergency and post-State of Emergency, from Q1 to Q5 [State of Emergency, PR(North | Q5)=2.26 (95% CI 1.63–3.14) and PR(LTV | Q5)=2.24 (95% CI 1.55–3.24); post-State of Emergency, PR(North | Q5)=3.72 (95% CI 2.54–5.45) and PR(LTV | Q5)=3.92 (95% CI 2.55–6.03)].

In the sensitivity analysis, adjusting for clinical factors led to a substantial decrease in degrees of freedom, and inconsistent results were observed regarding the risk of SARS-CoV-2 infection, as such, comorbidities were excluded from the multilevel analysis. A 98.0% concordance was observed between the occurrence of SARS-CoV-2 infection and residence parish, and the sensitivity analysis did not show differences in the direction and/or strength of the results depending on the parish information used.

## Discussion

People living in more socioeconomically deprived communities were found to have a higher risk of SARS-CoV-2 infection. This higher risk follows an increasing gradient from the least to the most deprived groups, emerging during the State of Emergency and intensifying following the end of the State of Emergency period. These results are consistent with those described in the literature.^4^^,^^15^^,^^23^^,^^24^ Furthermore, to the best of our knowledge, this is the first study to show a positive association between socioeconomic deprivation and the risk of SARS-CoV-2 infection involving all suspected cases of COVID-19 identified in a country, under the same political, social and organizational context, despite regional and local differences.

Demographic factors (sex and age) are independent features of infection, which have been described in previous publications, with males having a higher risk of SARS-CoV-2 infection though the total number of females suspected of having SARS-CoV-2 infection was higher in all quintiles, possibly because of more work exposure in social and health care jobs, and hence being tested more often.^4^^,^^25–27^ Geographical factors (population density and typologies of urban areas) increased the magnitude of the observed associations, due to the contextual variation related to transmission dynamics and susceptible individuals.^25^^,^^28^ Furthermore, the time between symptom onset and diagnosis was similar between socioeconomic groups, which may be related to tele-mechanisms for access healthcare (e.g. Portuguese health system hotline).^28^^,^^29^

The pre-State of Emergency was the shortest period and concentrated the smallest number of cases. Restrictive measures were not in place and there was a more specific case definition (epidemiological link with a confirmed case or geographical area with active community transmission). During this period, cases were mainly imported (male individuals of working age who had travelled internationally), which may explain the fact that there were no statistical differences between socioeconomic groups. In the following phases (State of Emergency and post-State of Emergency), when there was already active community transmission, social and physical distancing procedures were implemented. Therefore, an increasing gradient of the SARS-CoV-2 infection risk from the lowest to the highest level of socioeconomic deprivation was seen. The differences observed between socioeconomic groups raise the hypothesis that the most deprived population groups tend to work in essential sectors (infrastructures and cleaning maintenance, energy and food sectors, among others), have worse housing conditions (e.g. overcrowding, thus reducing the ability to isolate), use public transportation, and have a greater inability to adopt individual protection measures, due to the lack of economic support and lower literacy levels.^7^^,^^11^^,^^30^^,^^31^ Finally, these groups also have higher food insecurity, strengthening the negative synergistic effect between communicable diseases and nutritional status.^28^ Likewise, risk perception among individuals from most deprived areas might influence inappropriate and unsafe behaviours according to the epidemiological situation.^3^^,^^14^^,^^30^ In the presence of active community transmission or in clusters, the most deprived appear to be the most affected groups.^4^^,^^32^ This transmission dynamic may contribute to the maintenance of transmission chains that occur in the community, with an impact on the reproducibility number of SARS-CoV-2 infection.^33^^,^^34^ These outcomes may have contributed to the widening gap observed between socioeconomic groups.

### Strengths and limitations

The strengths and limitations of the study are related to data quality, exposure and effect variables, and contextual factors. Additionally, regarding asymptomatic disease cases, the dynamics of SARS-CoV-2 infection transmission is not yet fully understood, so all cases with positive laboratory results were subjected to the same preventive measures and control, regardless of their clinical manifestations.^26^^,^^35^ Study design limitations include the lack of temporal relation between exposure and outcome, and the use of secondary data.

Other potential weaknesses are related to the under representation of cases not captured by the formal epidemiological surveillance system, due to barriers in healthcare access. This may have resulted in underestimated socioeconomic differences considering the lack of access to healthcare in the most deprived populations. Also, underdiagnosis of asymptomatic cases may lead to an under representation, though the occurrence of this clinical course of infection also depends on other biological and environmental factors that are expected to be equally affected by socioeconomic factors.^36^^,^^37^ However, the SINAVE^®^ information system is representative of the Portuguese population, it has been running electronically for 5 years, and includes public, private and social sectors.^38^ In this way, the variables defined for the study are those that demonstrate greater completeness and quality, guaranteeing the accuracy and validity of the information. Nevertheless, data quality changed overtime during the epidemic response.

The exposure variable (socioeconomic deprivation) was built for the Portuguese population, validated with mortality indicators, and an individual’s allocation to a quintile was carried out through the parish where the infection occurred. Limitations can include parish misallocation, potential ecological fallacy (occurs when inference of individual socioeconomic status are incorrectly deduced from the socioeconomic level of the parish where the individual was infected) and variable construction using data collected in 2011. However, while parishes are the smallest geographical unit, there is variability in the EDI distribution within parishes, which may wash-away differences and lead to underestimated associations.^28^ The effect variable was collected through the laboratory result of SARS-CoV-2 infection, showing high precision and validity due to mandatory notification. However, sensitivity, time and method of collection, as well as laboratory analysis can be limitations.^26^^,^^36^ At last, this study did not analyse an association between disease severity and socioeconomic deprivation—which is poorly studied and the evidence thus far is inconsistent.^39^^,^^40^ As such, further research is needed on the association between socioeconomic deprivation and SARS-CoV-2 health outcomes.

Finally, the contextual factors are characterized by the implementation of exceptional measures, and by differences both in demand and in the provision of health services in Portugal.^3^^,^^14^ Therefore, the change in the health needs and the implemented policies, potentially interfered with the normal healthcare functioning observed until then.

## Conclusions

This study generated evidence on the effect of socioeconomic deprivation on the risk of SARS-CoV-2 infection, showing that health inequalities throughout the epidemic increased when social distancing policies eased. This adds to the already known information that the most deprived population groups tended to be more exposed to the infection as they work in essential sectors, have worse housing conditions (which can facilitate transmission and does not allow for the implementation of quarantine or isolation if necessary), use public transportation more often and have a higher inability to adopt individual protection measures, due to the lack of economic support and lower literacy levels.^7^^,^^11^^,^^30^

In epidemic situations of active community transmission or by clusters, the most deprived socioeconomic population groups appear to be the most affected segments, sustaining transmission chains in the community. Social determinants contribute the most to health inequalities in a population, to which Portugal is no different. Therefore, empowering individuals, improving health literacy, implementing participated and integrative social policies, and promoting healthy environments, should be addressed in an integrated, systemic and holistic view, not only during the response to the COVID-19 pandemic but beyond.

## Supplementary data

Supplementary data are available at *EURPUB* online.

## Supplementary Material

ckab192_Supplementary_Data
