# Supplementary material for: Community socioeconomic deprivation and SARS-CoV-2 infection risk: findings from Portugal
Source: Eur J Public Health. 2021 Nov 11;32(1):145–50. doi: 10.1093/eurpub/ckab192 (PMC8689925; doi:10.1093/eurpub/ckab192)
Supplement: ckab192_Supplementary_Data [file ckab192_supplementary_data.zip › ejph-2021-04-om-0433-File004.docx]

| **Model 0** | **Model 3** |
| --- | --- |
| 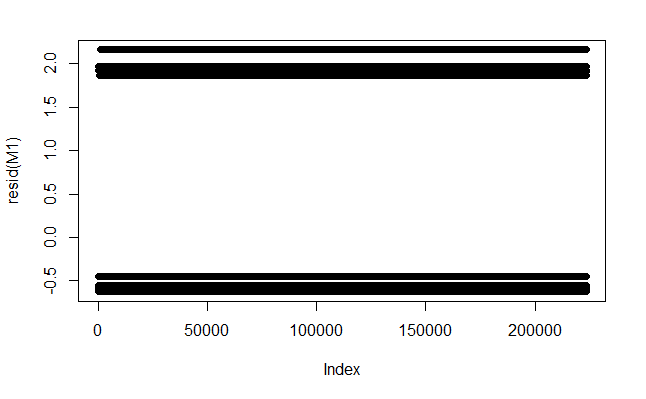 | 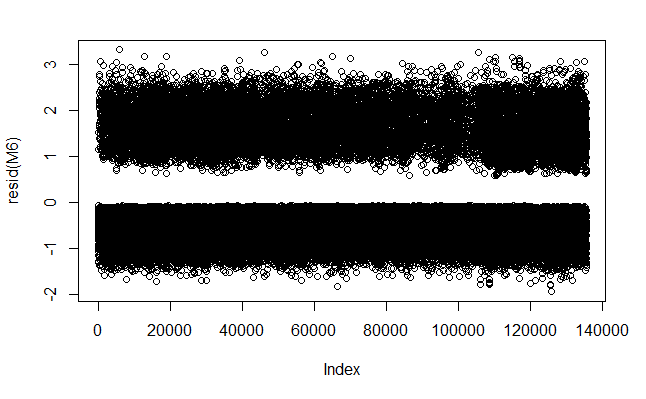 |
| **Model 1** | **Model 4** |
| 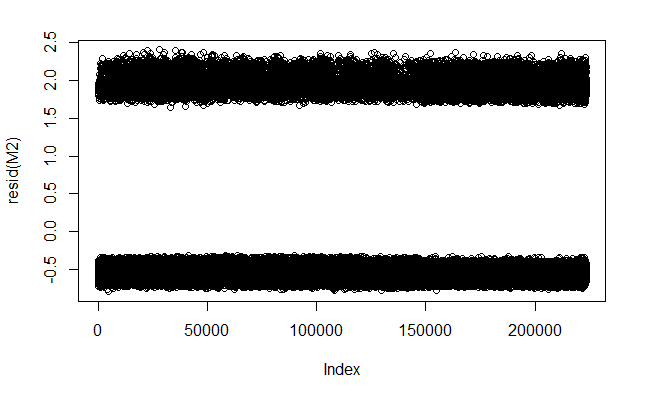 | 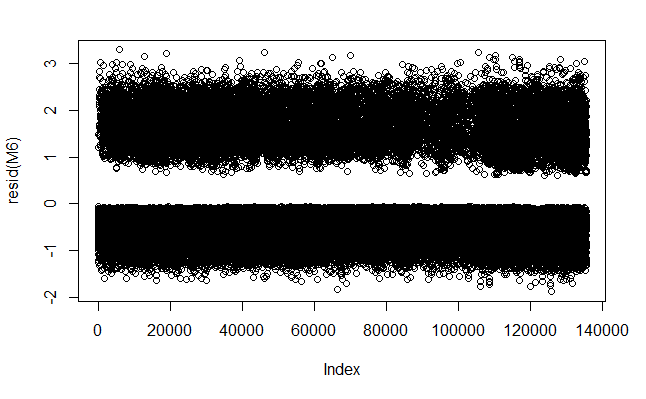 |
| **Model 2** |  |
| **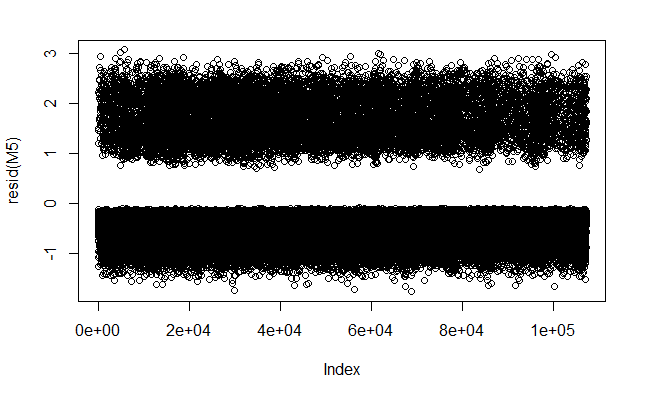** |  |

**Figure S1** | Residuals distribution of multilevel models

**Table S1 |** Degrees of freedom, residuals deviance and Akaike Information Criterion (AIC) of multilevel models

| **Model** | **Degrees of freedom** | **Residuals deviance** | **AIC** |
| --- | --- | --- | --- |
| 0 | 223 328 | 184 920 | 184 930 |
| 1 | 223 326 | 184496 | 184 510 |
| 2 | 223 324 | 166 911 | 166 929 |
| 3 | 223 323 | 166 524 | 166 544 |

**Figure S2 |** Residuals deviance of multilevel models

| **Model 1** | **Model 3** |
| --- | --- |
| 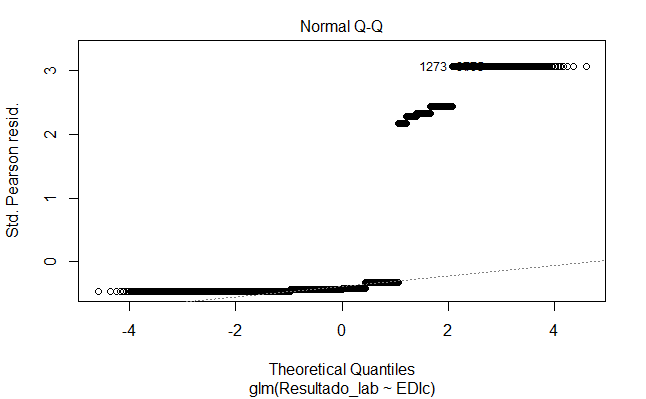 | 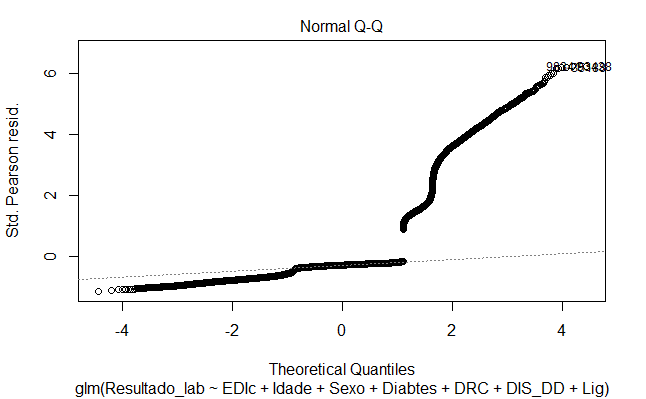 |
| **Model 2** | **Model 4** |
| 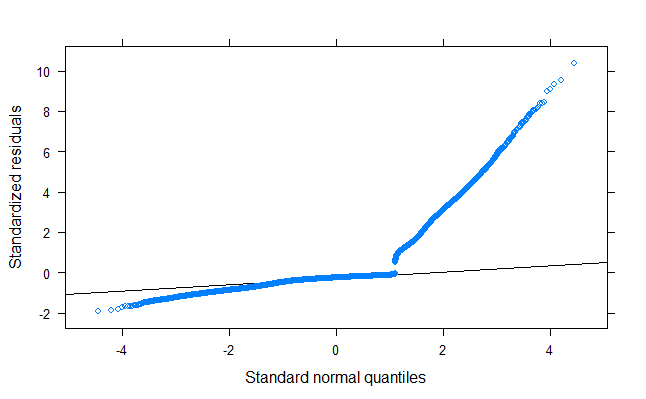 | 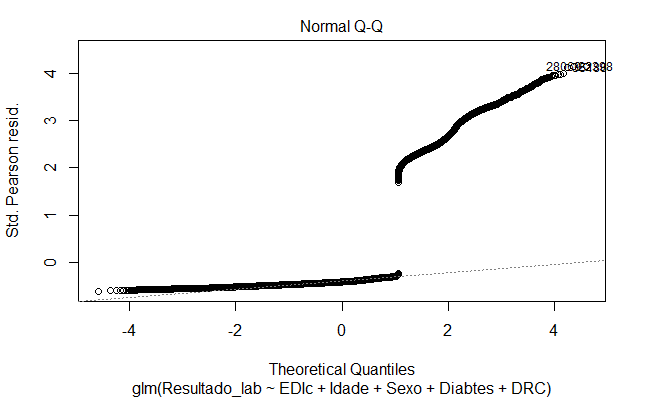 |
