# Supplementary material for: Community socioeconomic deprivation and SARS-CoV-2 infection risk: findings from Portugal
Source: Eur J Public Health. 2021 Nov 11;32(1):145–50. doi: 10.1093/eurpub/ckab192 (PMC8689925; doi:10.1093/eurpub/ckab192)
Supplement: ckab192_Supplementary_Data [file ckab192_supplementary_data.zip › ejph-2021-04-om-0433-File005.docx]

**Table S2 |** Degrees of freedom, residuals deviance and Akaike Information Criterion (AIC) of multilevel models, by response phase

| **Model** | **Degrees of freedom** | **Residuals deviance** | **AIC** |
| --- | --- | --- | --- |
| Pre-State of Emergency | 22,880 | 21,566 | 21,586 |
| State of Emergency | 121,035 | 89,358 | 89,278 |
| Post-State of Emergency | 79,388 | 51,247 | 51,267 |

|  | **Model** | **Degrees of freedom** | **Residuals deviance** | **AIC** |
| --- | --- | --- | --- | --- |
| North | Pre-State of Emergency | 11,023 | 11,880 | 11,898 |
|  | State of Emergency | 53,407 | 47,482 | 47,800 |
|  | Post-State of Emergency | 24,936 | 11,829 | 11,847 |
|  | Total | 89,383 | 71,793 | 71,813 |
|  | **Model** | **Degrees of freedom** | **Residuals deviance** | **AIC** |
| Lisbon and Tagus Valley | Pre-State of Emergency | 7,147 | 5,860 | 5,878 |
|  | State of Emergency | 38,433 | 26,576 | 26,594 |
|  | Post-State of Emergency | 34,504 | 32,909 | 32,927 |
|  | Total | 80,101 | 65,658 | 65,678 |

**Table S3 |** Degrees of freedom, residuals deviance and Akaike Information Criterion (AIC) of multilevel models, by response phase and both North and Lisbon and Tagus Valley health regions
