# Supplementary material for: Community socioeconomic deprivation and SARS-CoV-2 infection risk: findings from Portugal
Source: Eur J Public Health. 2021 Nov 11;32(1):145–50. doi: 10.1093/eurpub/ckab192 (PMC8689925; doi:10.1093/eurpub/ckab192)
Supplement: ckab192_Supplementary_Data [file ckab192_supplementary_data.zip › ejph-2021-04-om-0433-File006.docx]

**Table S4** | Frequencies and unadjusted prevalence ratios (PR) of geodemographic, clinical and epidemiological factors, by SARS-CoV-2 infection laboratory result

| SARS-CoV-2 laboratory result | Positive  (n = 32,784) | Negative  (n = 190,549) | PR [CI 95%] |
| --- | --- | --- | --- |
| **Geodemographic factors** |  |  |  |
| Age (years)  Mean (IQR) | 48 (32-64) | 46 (29-65) | 1.00 [1.00-1.01]* |
| Sex [n (%)]  Female  Male | 18,659 (56.9)  14,125 (43.1) | 109,915 (57.7)  80,634 (42.3) | (ref)  1.07 [1.02-1.12]* |
| Health region [n (%)]  North  Centre  LTV  LTV  *Alentejo*  *Algarve*  *Açores*  *Madeira* | 73,900 (47.3)  26,744 (10.9)  67,194 (39.4)  10,642 (0.8),  9,940 (1.1)  652 (0.3)  1,477, 0.3) | 15,493 (38.8)  3,566 (14.0)  12,917 (35.3)  266 (5.6)  365 (5.2)  93 (0.3)  84 (0.8) | (ref)  0.81 [0.75-0.87]*  0.72 [0.68-0.76]*  0.24 [0.19-0.29]*  0.22 [0.19-0.26]*  1.35 [0.91-1.94]  0.57 [0.37-0.85]* |
| Urban areas [n (%)]  PUA  MUA  PRA | 137,424 (84.0)  25,683 (9.9)  27,442 (6.0) | 27,553 (72.1)  3,258 (13.5)  1,983 (14.4) | (ref)  0.90 [0.83-0.97]*  0.60 [0.55-0.66]* |
| Population density (habitants/km^2^)  Median (IQR) | 1,802.6  (448.6-4,820.7) | 616.9  (150.8-2,734.6) | 1.00 [1.00-1.00]* |
| **Clinical factors** |  |  |  |
| Comorbidities [n (%)]  No  Yes | 10,221 (57.9)  7,437 (42.1) | 69,051 (58.1)  49,846 (41.9) | (ref)  1.01 [0.98-1.04] |
| **Epidemiological factors** |  |  |  |
| Delay (days)  Median (IQR) | 3.0 (2.0-7.0) | 2.0 (1.0-6.0) | 1.00 (0.99-1.01) |
| Epidemiology link [n (%)]  No  Yes | 6,735 (33.8)  13,164 (66.2) | 86,027 (74.4)  29,560 (25.6) | (ref)  5.69 [5.51-5.87]* |
| Response phase [n (%)]  Pre-State of Emergency  State of Emergency  Post-State of Emergency | 4,729 (14.4)  17,979 (54.8)  10,076 (30.7) | 18,161 (9.5)  103,066 (54.1)  69,322 (36.4) | (ref)  0.67 [0.65-0.69]*  0.56 [0.54-0.58]* |

Footnote: CI (confidence interval), IQR (interquartile range), LTV (Lisbon and Tagus Valley) PUA (predominantly urban areas), MUA (mainly urban areas) and PRA (predominantly rural areas), *p<0.05
