# Supplementary material for: Community socioeconomic deprivation and SARS-CoV-2 infection risk: findings from Portugal
Source: Eur J Public Health. 2021 Nov 11;32(1):145–50. doi: 10.1093/eurpub/ckab192 (PMC8689925; doi:10.1093/eurpub/ckab192)
Supplement: ckab192_Supplementary_Data [file ckab192_supplementary_data.zip › ejph-2021-04-om-0433-File007.docx]

**Table S5** | Adjusted (aPR) and unadjusted (PR) prevalent ratios between socioeconomic deprivation, by quintiles, and risk of SARS-CoV-2 infection

|  | **Model 0** | **Model 1** | **Model 2** | **Model 3** |
| --- | --- | --- | --- | --- |
|  | PR [CI 95%] | aPR [CI 95%] | aPR [CI 95%] | aPR [CI 95%] |
| Socioeconomic deprivation (quintiles)  Q1 (least deprived, ref)  Q2  Q3  Q4  Q5 | 1.57 [1.51-1.64]*  1.72 [1.65-1.79]*  1.80 [1.73-1.87]*  1.99 [1.92-2.07]* | 1.59 [1.53-1.66]*  1.76 [1.69-1.84]*  1.85 [1.78-1.93]*  2.06 [1.98-2.15]* | 1.25 [1.08-1.45]*  1.24 [1.05-1.46]*  1.29 [1.09-1.53]*  1.21 [0.99-1.48] | 1.37 [1.19-1.58]*  1.48 [1.26-1.73]*  1.73 [1.47-2.04]*  2.24 [1.83-2.75]* |

Footnotes:

Model 1: adjusted for age and sex

Model 2: M1 + urban areas and population density

Model 3: M2 + health region

*p<0.05
