# Supplementary material for: Community socioeconomic deprivation and SARS-CoV-2 infection risk: findings from Portugal
Source: Eur J Public Health. 2021 Nov 11;32(1):145–50. doi: 10.1093/eurpub/ckab192 (PMC8689925; doi:10.1093/eurpub/ckab192)
Supplement: ckab192_Supplementary_Data [file ckab192_supplementary_data.zip › ejph-2021-04-om-0433-File008.docx]

**Table S6 |** Adjusted prevalence ratios (aPR) between socioeconomic deprivation, by quintiles and total, and risk of SARS-CoV-2 infection, by response phase

|  | **Pre-State of Emergency** | **State of Emergency** | **Post-State of Emergency** |
| --- | --- | --- | --- |
|  | aPR [CI 95%] | aPR [CI 95%] | aPR [CI 95%] |
| Socioeconomic deprivation (quintiles)  Q1 (least deprived, ref)  Q2  Q3  Q4  Q5 | 1.20 [0.99-1.45]  1.26 [1.03-1.54]*  1.25 [1.01-1.54]*  1.43 [1.10-1.86]* | 1.36 [1.17-1.59]*  1.54 [1.30-1.83]*  1.76 [1.48-2.11]*  2.09 [1.67-2.62]* | 1.51 [1.25-1.83]*  1.49 [1.21-1.85]*  1.89 [1.52-2.36]*  3.43 [2.66-4.44]* |
