# Supplementary material for: Community socioeconomic deprivation and SARS-CoV-2 infection risk: findings from Portugal
Source: Eur J Public Health. 2021 Nov 11;32(1):145–50. doi: 10.1093/eurpub/ckab192 (PMC8689925; doi:10.1093/eurpub/ckab192)
Supplement: ckab192_Supplementary_Data [file ckab192_supplementary_data.zip › ejph-2021-04-om-0433-File009.docx]

|  | **Pre-State of Emergency** | | **State of Emergency** | | **Post-State of Emergency** | | **Total** | |
| --- | --- | --- | --- | --- | --- | --- | --- | --- |
|  | **North** | **LTV** | **North** | **LTV** | **North** | **LTV** | **North** | **LTV** |
|  | aPR [CI 95%] | | aPR [CI 95%] | | aPR [CI 95%] | | aPR [CI 95%] | |
| Socioeconomic deprivation (quintiles)  Q1 (least deprived, ref)  Q2  Q3  Q4  Q5 | 1.27 [0.98-1.65]  1.42 [1.09-1.85]*  1.24 [0.94-1.63]  1.23 [0.79-1.90] | 1.18 [0.74-1.89]  0.98 [0.59-1.63]  1.34 [0.83-2.18]  1.47 [0.91-2.38] | 1.54 [1.26-1.90]*  1.87 [1.50-2.33]*  2.09 [1.67-2.61]*  2.26 [1.63-3.14]* | 1.21 [0.86-1.72]  0.91 [0.62-1.33]  1.54 [1.06-2.22]*  2.24 [1.55-3.24]* | 1.85 [1.43-2.39]*  2.11 [1.61-2.76]*  2.42 [1.84-3.19]*  3.72 [2.54-5.45]* | 1.61 [1.08-2.41]*  1.41 [0.90-2.18]  2.10 [1.37-3.22]*  3.92 [2.55-6.03]* | 1.50 [1.25-1.80]*  1.78 [1.47-2.17]*  1.98 [1.62-2.42]*  2.30 [1.72-3.08]* | 1.55 [1.11-2.18]*  1.32 [0.91-1.91]  1.96 [1.36-2.82]*  3.05 [2.10-4.19]* |

**Table S7 |** Adjusted prevalence ratios (aPR) between socioeconomic deprivation, by quintiles and total, and risk of SARS-CoV-2 infection, by response phase and both North and Lisbon and Tagus Valley health regions

Footnote: aPR (adjusted prevalence ratios), CI (confidence intervals) and LTV (Lisbon and Tagus Valley), *p<0.05
